# Saikosaponin D Isolated from *Bupleurum falcatum* Inhibits Selectin-Mediated Cell Adhesion

**DOI:** 10.3390/molecules191220340

**Published:** 2014-12-04

**Authors:** Myoung-Jun Jang, Ye Sol Kim, Eun Young Bae, Tae-Seok Oh, Hwa-Jung Choi, Jung-Hee Lee, Hyun-Mee Oh, Seung Woong Lee

**Affiliations:** 1 Enviromental Agriculture Research Division, Gyeonggido Agricultural Reaearch and Extension Services(GARES), Hwaseong, Gyeonggi-do 445-784, Korea; E-Mail: jplant119@gmail.com; 2 Bioindustial Process Research Center, Korea Research Institute of Bioscience and Biotechnology (KRIBB), Jeongeup-si, Jeollabuk-do 580-185, Korea; E-Mail: yesolkim@kribb.re.kr; 3 LINC project group, University Industry Cooperation, Daejeon University, Daejeon 300-716, Korea; E-Mail: eunjs75@dju.kr; 4 Department of Plant Resources, Kongju National University, Yesan 340-702, Korea; E-Mail: ots1022@naver.com; 5 Department of Beauty Science, Kwangju Women’s University, Gwangju 506-713, Korea; E-Mails: rerived@kwu.ac.kr (H.-J.C.); jh4471@kwu.ac.kr (J.-H.L.); 6 Department of Chemistry, Mokwon University, Daejeon 302-729, Korea

**Keywords:** *Bupleurum falcatum* L., triterpenoid saponin, saikosaponin, selectin, cell adhesion molecules, anti-inflammatory agents

## Abstract

Three saikosaponins were isolated from the MeOH extract of the roots of *Bupleurum falcatum* L.: saikosaponins B3 (**1**); B4 (**2**); and D (**3**). Of the three, compound **3** inhibited the interaction of selectins (E, L, and P) and THP-1 cells with IC_50_ values of 1.8, 3.0 and 4.3 µM, respectively. Also, the aglycone structure **4** of compound **3** showed moderate inhibitory activity on L-selectin-mediated cell adhesion. From these results, we suspect that compound **3** isolated from *Bupleurum falcatum* roots would be a good candidate for therapeutic strategies to treat inflammation.

## 1. Introduction

The interactions between circulating leukocytes and vascular endothelial cells play an important role in the development of inflammatory diseases such as atherosclerosis, asthma, and rheumatoid arthritis [1,2]. Leukocyte trafficking is also an essential part of the immune response [3]. In general, cell to cell interactions are mediated by various cell adhesion molecules (ICAM-1, VCAM-1, and selectins), these ligands, cytokines, and chemokines. In particular, selectins are involved in the initial adhesive step in the recruitment and migration of immune cells to the inflammatory site [4,5]. The selectins have been categorized into three cell-surface glycoproteins: E-selectin, L-selectin, and P-selectin. The major ligand for P-selectin is P-selectin glycoprotein ligand-1 (PSGL-1, CD 162), constitutively expressed by leukocytes. It, together with CD44, can bind to E-selectin and L-selectin [6]. E-selectin and P-selectin are expressed on the cell-surface of endothelial cells by pro-inflammatory cytokines such as TNF-α, IL-1β, and IL-6 [7]. In contrast, L-selectin, which is present on leukocytes, is responsible for lymphocyte binding to endothelium and for the permeation of neutrophils in the inflammatory site stimulated by viruses and bacteria [8]. Selectins are also related to lymphocyte homing, chronic and acute inflammation processes including brain, lung, heart and skin inflammation, as well as cancer progression [1,2]. Therefore, blocking of selectins is considered a promising therapeutic approach for the prevention and treatment of inflammatory diseases, which are caused by the recruitment and infiltration of leukocytes.

*Bupleurum falcatum* L (*B*. *falcatum*, Apiaceae) is one of the most commonly used crude drugs in China and Korea. It was used as an important component of traditional oriental medicines for the treatment of chronic hepatitis and auto-immune diseases [9]. Saikosaponins, isolated from *B*. *falcatum*, are known to have numerous pharmacological activities including anti-inflammatory, anti-bacterial, anti-tumor, and anti-allergic activities [10,11,12]. We reported previously that the saikosaponins inhibited cell adhesion by VCAM-1/VLA-4 [13]. In the current study, we investigated the potent effect of saikosaponins, isolated from the MeOH extract of *B*. *falcatum*, on selectin-mediated cell adhesion.

## 2. Results and Discussion

### 2.1. Isolation and Identification of Compounds **1**–**3**

**Figure 1:**
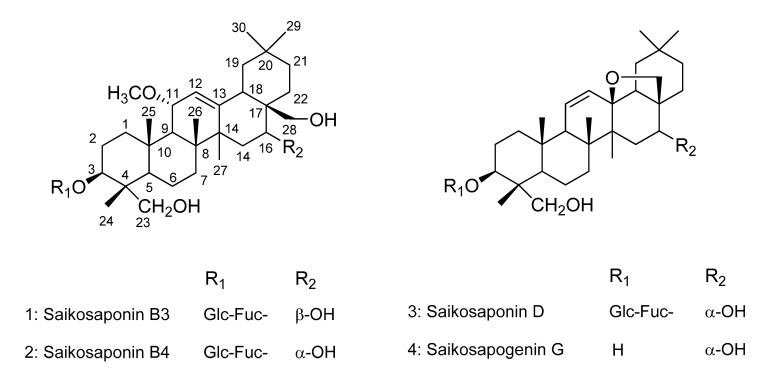
Chemical structures of compounds **1**–**4**.

During an investigation of selectin-mediated cell adhesion inhibitors obtained from natural sources, the MeOH extract of the roots of *B. falcatum* were observed to inhibit the interaction of selectin and THP-1 cells (data not shown). To isolate the active compounds with an inhibitory effect on the selectin/PSGL-1 interaction, the MeOH extract of the roots of *B. falcatum* was suspended in water and partitioned with CHCl_3_. The CHCl_3_–soluble fraction was separated on silica gel and ODS open-column chromatography, and then subjected to semi-preparative HPLC to yield compounds **1**–**3**. Compound **3** was treated with an alcoholic alkali metal solution to preserve the epoxy moiety in the aglycone form **4** [14], and subsequently subjected to semi-preparative HPLC to yield **4**. As shown in Figure 1, the structures were confirmed as saikosaponins B3 (**1**), B4 (**2**), and D (**3**) and also saikosapogenin G (**4**) through NMR and MS spectra analyses, and comparison with published data [14,15,16,17].

### 2.2. Cell Adhesion Inhibitory Activities

#### 2.2.1. Inhibitory Effects of Compounds **1**–**4** on Selectins (E, L, and P)-Mediated Cell Adhesion Activities *in Vitro*

**Figure 2:**
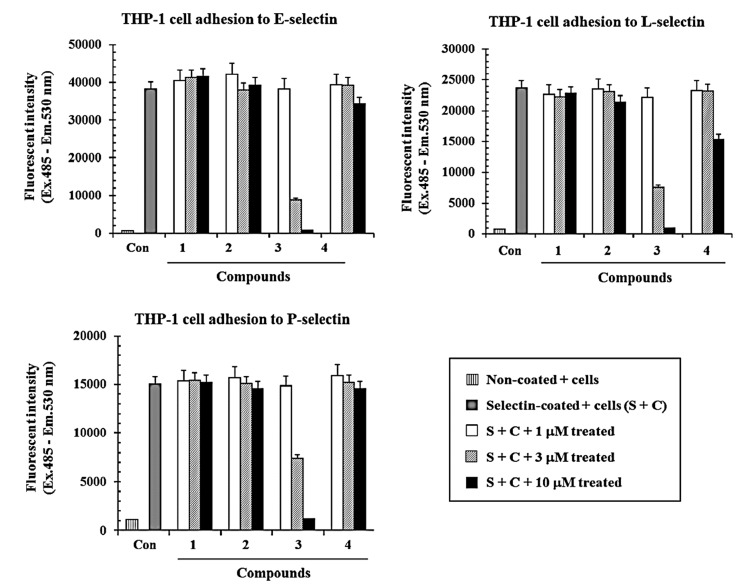
Inhibitory effects of compounds **1**–**4** on the cell adhesion on E-, L-, P-selectin. BCECF-AM-labeled THP-1 cells and test compounds **1**–**4** were added to 96-well plates coated with E-selectin, L-selectin, and P-selectin, respectively. After incubation for 1 h at 37 °C, cells were dissolved in 1% Triton X-100 in PBS, and fluorescent intensity was measured as described in the Experimental section. The data are presented as the means of three independent experiments performed in duplicate.

The effects of compounds **1**–**3** on the binding between THP-1 cells and recombinant E-selectin, L-selectin, and P-selectin (R&D Systems, Minneapolis, MN, USA), were tested using a modified ELISA method [13]. Briefly, fluorescence-labeled THP-1 cells were added to selectin-coated plates in the presence or absence of the compounds for 1 h at 37 °C. After then, fluorescent intensity of THP-1 cells adhered on selectin were measured. Compound **3** inhibited direct binding between E-selectin and THP-1 cells in a dose-dependent manner, with an IC_50_ value of 1.8 µM (Figure 2). Compounds **1** and **2** did not show any inhibitory activity in the cell adhesion assay (<10% at 10 µM, Figure 2). These compounds were examined further to determine if they could affect binding between the cells and other selectins such as L-selectin and P-selectin. Compound **3** also showed inhibitory activity on cell adhesion mediated by L-selectin and P-selectin in a dose dependent manner with an IC_50_ value of 3.0 µM and 4.3 µM (Figure 2), respectively. However, the other compounds (**1** and **2**) showed no inhibitory effects on the cell adhesion similar to the result with E-selectin (<10% at 10 µM, Figure 2). THP-1 or HL-60 cells did not attach on non-coated plate while the cells adhered on selectin-coated plate, suggesting that it is a specific interaction mediated by selectin.

**Figure 3:**
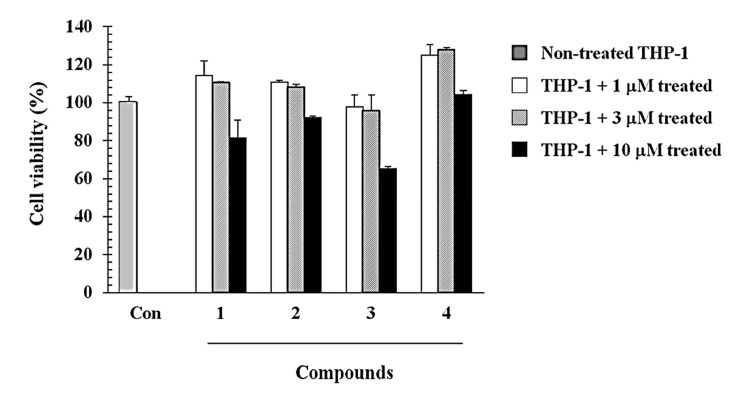
Effect of compounds **1**–**4** on THP-1 cell viability. Cells were incubated with the indicated concentrations of compounds **1**–**4** for 48 h. The viability was determined by the MTT-based cytotoxicity assay. The % viability was calculated as follows: (absorbance for compound treated cells / absorbance for untreated cells) × 100. Data are presented as the mean ± SE (*n* = 3).

In addition, as compound **3** lacked cytotoxic effects on THP-1 cells at the concentrations employed in this study, it was investigated even further to confirm that the inhibitory activity was not due to cytotoxic action. As shown in Figure 3, compound **3** exhibited 65% cell viability at the concentration of 10 µM, and did not show any cytotoxicity at the other concentrations used in this study.

As shown in Figure 1 and Figure 2, compounds **1** and **2**, which possess methoxy and hydroxymethyl groups in addition to two sugar moieties, had no inhibitory effects on the direct binding between the selectins and THP-1 cells. However, compound **3**, containing an epoxy group and two sugar moieties, showed potent inhibitory activities compared to compounds **1** and **2** in this assay. This result suggested that the presence of the epoxy group may affect cell adhesion inhibitory activity. In particular, we tested whether the aglycone **4**, which lacked the two sugar moieties of compound **3**, showed the same inhibitory activity in the binding between selectins (E, L, and P) and THP-1 cells. Compound **4** showed 40% inhibitory activity in the cell adhesion assay with L-selectin at a concentration of 10 µM, and it still had moderate activity compared with compound **3**. However, it only showed 20% and 10% inhibitory activities at a concentration of 10 µM in assays with E-selectin and P-selectin, respectively. This result suggested that the presence of the sugar moieties could affect the cell adhesion inhibitory activity.

#### 2.2.2. Compound **3** Inhibits Monocyte Adhesion onto Endothelial Cells Activated by TNF-α

**Figure 4:**
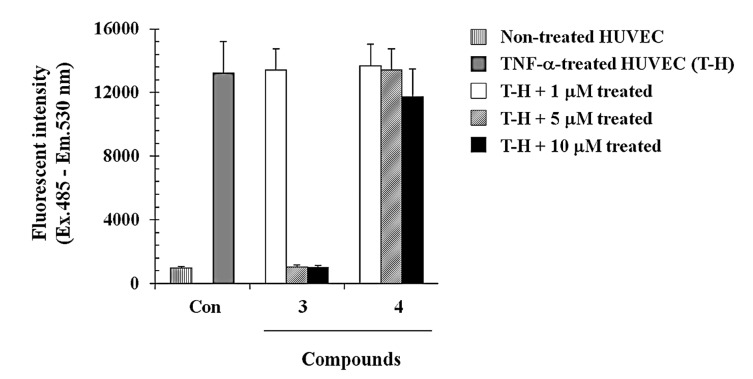
Inhibitory effects of compounds **3** and **4** on THP-1 monocyte adhesion to HUVECs. HUVECs were incubated with 5 ng/mL TNF-α in the absence or presence of compounds **3** and **4** (1, 5, and 10 μM). Fluorescence-labeled THP-1 cells were added to the HUVECs and allowed to incubate for 1 h. The fluorescent intensity was measured as described in the Experimental Section. The data are presented as the means of three independent experiments performed in duplicate.

We further investigated whether compounds **3** and **4** were able to modulate cell to cell interaction using a monocyte-endothelial cell adhesion assay. HUVEC cells were stimulated with TNF-α (5 ng/mL) for 6 h prior to the addition of THP-1 cells. THP-1 cells were incubated with or without compounds **3** and **4** at various concentrations (final concentrations of 1, 5, and 10 µM) for 30 min, and then added onto HUVEC cells activated with TNF-α. As shown in Figure 4, compound **3** significantly inhibited the THP-1 adhesion to the HUVECs monolayer in a concentration dependent manner. The inhibition of monocyte-endothelial cell adhesion was showed as more than 95% at the concentrations of 5 µM and 10 µM (Figure 4). However, compound **4** failed to inhibit the adhesion as shown in Figure 4, suggesting that the presence of the sugar moieties could affect the cell adhesion inhibitory activity.

#### 2.2.3. Compound **3** Inhibits TNF-α-Induced Cell Adhesion Molecule Expression in Monocytes

**Figure 5:**
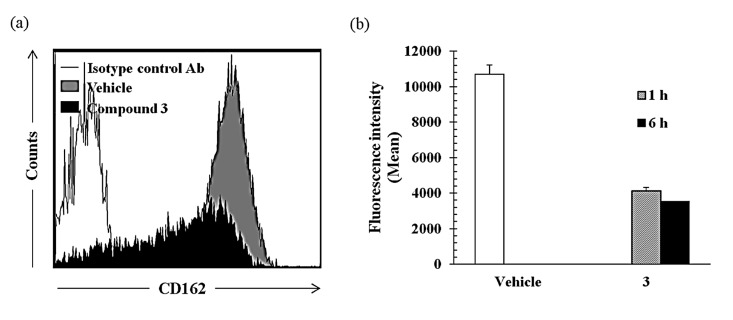
Compound **3** inhibits the expression of P-selectin ligand (CD162) in THP-1 cells. THP-1 cells were incubated with compound **3** (30 μM) for 1 and 6 h and then CD162 expression was analyzed by FACS. (**a**) An overlay FACS histogram of THP-1 cells stimulated with compound **3** for 6 h and (**b**) mean fluorescence intensity were shown.

Compound **3** showed potent inhibitory activities on the cell adhesion assay using three recombinant proteins (E-selectin, L-selectin, and P-selectin) and monocytes. This can be a means that compound **3** inhibits direct binding between selectins and their ligands expressed on monocytes. Alternatively, this compound can be inhibits monocyte adhesion through the reduction of the expression of selectin ligand. Therefore, we investigated here the effect of compound **3** on expression of P-selectin ligand (PSGL-1/CD162) in THP-1 cells by FACS analysis (Figure 5). Isotype control antibody used for control. CD162 expression was significantly reduced by the treatment with compound **3** relative to vehicle in a time dependent manner (40% and 35% of control at 30 μM for 1 h and 6 h, respectively), it suggesting this compound partially regulate the cell adhesion through the regulation of the selectin ligand expression.

## 3. Experimental Section

### 3.1. Plant Material, Extraction and Isolation

The *B. falcatum* roots were purchased at an herbal market in Daejeon, Korea. A voucher specimen (PBC-247A) was deposited in the Korea Plant Extract Bank, at the Korea Research Institute of Bioscience and Biotechnology. The dried roots of the *B. falcatum* (2.4 kg) were extracted with MeOH (10 L) for 7 days at room temperature. The MeOH extract was evaporated *in vacuo*, yielding a residue (435 g). The residue was suspended in distilled water (2 L) and extracted with CHCl_3_ (8 L). The CHCl_3_ layers were then evaporated *in vacuo*, with the resulting extract (180 g) subjected to silica gel (230–400 mesh, 1.8 kg, Merck KGaA, Darmstadt, Germany) column chromatography using a gradient of CHCl_3_–MeOH (100:0, 90:1, 70:1, 50:1, 30:1, 15:1, 5:1, 1:1; each 3.0 L, v/v). Based on the TLC profile, 19 fractions (F1–F19) were collected, with each fraction monitored by an *in vitro* cell adhesion inhibitory assay. Fraction F18 (15 g) showed the highest inhibitory effect and was subjected to reverse-phase column chromatography (300 g), then eluted with MeOH–H_2_O (70:30, 80:20, 90:10, 100:0; each 2.0 L, v/v), to yield 11 sub-fractions (F18-1–F18-11), based on the TLC profile. In addition, F18-3 (680 mg) was successively separated by semi-preparative HPLC (MeOH–H_2_O, 65:35, v/v), yielding **1** (19 mg), and **2** (18 mg). Fraction F18-4 (687 mg) was successively separated by semi-preparative HPLC (YMC-pack ODS-H80, 4 µm, 250 × 20 mm, flow rate 5.0 mL/min) and elution with MeOH–H_2_O (65:35, v/v), yielding **3** (18 mg). Compound **3** (70 mg) was dissolved in butanol (10 mL), followed by addition of sodium metal (350 mg). This mixture was allowed to react for 6 h at 80 °C. Water was then added to stop the reaction. The butanol layer was washed with water three times and evaporated to dryness. The residue was then separated by semi-preparative HPLC (MeOH–H_2_O, 75:25, v/v), yielding **4** (15 mg).

### 3.2. Cell Adhesion to E-Selectin, L-Selectin, and P-Selectin

The Human acute monocytic leukemia cell line THP-1 was maintained in RPMI 1640, supplemented with 10% fetal calf serum, 100 U/mL penicillin and 100 μg/mL streptomycin sulfate. These cell lines were maintained at 37 °C in a humidified incubator containing 5% CO_2_. An adhesion assay using soluble E-selectin, L-selectin, and P-selectin and THP-1 cells was performed as described previously with slight modifications. 96-well plates were coated with 100 µL of recombinant E-selectin, L-selectin, and P-selectin (R&D systems) at a concentration of 5 µg/mL in PBS at 37 °C for 3–4 h. The wells were then washed twice with PBS and blocked by addition of 200 µL of PBS with 1% BSA and incubation for 1 h at room temperature. For fluorescent labeling of THP-1 cells, 1 × 10^6^ cells washed once in RPMI 1640 were re-suspended in 12 mL of RPMI 1640 containing 2 µM BCECF-AM (2',7'-bis-2-carboxyethyl-5-(6)-carboxyfluorescein acetoxymethyl ester), incubated at 37 °C for 1 h, and washed once with RPMI 1640/0.1% fetal bovine serum. Next, fluorescent-labeled THP-1 cells and 5 µL of compounds **1**–**4** were added to each well. The plates were incubated for 1 h at 37°C and the wells gently washed once with RPMI 1640/0.1% fetal bovine serum. Fluorescent intensity was measured with a fluorescent plate reader (FL×800TM, BioTek^®^ Instruments, Inc., Winooski, Vermont, USA) at an excitation wavelength of 485 nm and an emission wavelength of 530 nm.

### 3.3. Cell Adhesion to HUVEC

HUVECs in 150 µL complete growth medium were seeded onto 96-well plates at 1 × 10^4^ cells per well and incubated to conﬂuency overnight at 37 °C. Next, the HUVECs monolayer was stimulated with 5 ng/mL TNF-α (Sigma-aldrich, St. Louis, Missouri, USA) for 4 h. Then, fluorescent-labeled THP-1 cells and 5 µL of compound **3** were then added to each well. The plates were incubated for 1 h at 37 °C and the wells were gently washed once with RPMI 1640/0.1% fetal bovine serum. Fluorescent intensity was measured with a fluorescent plate reader at an excitation wavelength of 485 nm and an emission wavelength of 530 nm.

### 3.4. Cell Adhesion Molecule Expression on THP-1 Cells

The cell surface expression of P-selectin on THP-1 cells was further confirmed by flow cytometry. Briefly, THP-1 cells were incubated with or without compound **3** for 2 h followed by TNF-α (5 ng/mL). P-selectin was measured 4 h after TNF-α treatment. The cells were washed with PBS and dislodged, then incubated with anti-human CD162 (PSGL-1) or control IgG antibody (1.0 mg/10^6^ cells, 30 min, 4 °C). After incubation, the cells were washed with PBS and stained with FITC conjugated goat anti-human IgG for 30 min at 4 °C. Finally, the cells were fixed with 1.0% paraformaldehyde and analyzed for the expression of cell adhesion molecules using a flow cytometer (FACSArial II, BD Biosciences, San Jose, CA, USA). For each sample, 20,000 events were acquired. Analysis was carried out using FACSDiva software Version 6.1.2 (BD Biosciences). The auto-fluorescence intensity was subtracted from the treated conditions. Mean fluorescence intensity was estimated from three independent experiments and bar diagrams were plotted.

### 3.5. Cell Viability Assay

Cell viability was assessed by morphology and by reduction of the tetrazolium salt (MTT) as previously described [18]. Briefly, the THP-1 cells (2 × 10^5^ cells/well) and various concentrations of compounds **1**–**4** were added to the 96-well plates, incubated for 48 h at 37°C, and 5 µL of MTT solution (5 mg/mL in PBS) was added to each well of the 96-well plates. After incubation for 4 h at 37 °C, the absorbance was measured at 540 nm using a microplate reader (Bio-Rad, Hercules, CA, USA) with the reference absorbance at 650 nm.

## 4. Conclusions

In conclusion, three saikosaponins **1**–**3** were isolated from the MeOH extract of *Bupleurum falcatum* roots. Among them, compound **3** showed the highest potency in the assay system using recombinant proteins (E-selectin, L-selectin, and P-selectin). The aglycone structure **4** of compound **3** showed mild inhibitory activity on cell adhesion assay using L-selectin. In particular, compound **3** successfully inhibited monocyte adhesion to endothelial cells and reduce the expression of P-selectin ligand on THP-1 cells. These results could be useful for the design of new cell adhesion inhibitors for anti-inflammatory agents.
